# Strand bias in complementary single-nucleotide polymorphisms of transcribed human sequences: evidence for functional effects of synonymous polymorphisms

**DOI:** 10.1186/1471-2164-7-213

**Published:** 2006-08-17

**Authors:** Hui-Qi Qu, Steve G Lawrence, Fan Guo, Jacek Majewski, Constantin Polychronakos

**Affiliations:** 1 Endocrine Genetics Laboratory, The McGill University Health Center (Montreal Children's Hospital), Montréal, Québec, Canada; 2 Department of Human Genetics, McGill University, Montréal, Québec, Canada; 3 Department of Pediatrics, The McGill University Health Center (Montreal Children's Hospital), 2300 Tupper, Montréal, Québec H3H 1P3, Canada

## Abstract

**Background:**

Complementary single-nucleotide polymorphisms (SNPs) may not be distributed equally between two DNA strands if the strands are functionally distinct, such as in transcribed genes. In introns, an excess of A↔G over the complementary C↔T substitutions had previously been found and attributed to transcription-coupled repair (TCR), demonstrating the valuable functional clues that can be obtained by studying such asymmetry. Here we studied asymmetry of human synonymous SNPs (sSNPs) in the fourfold degenerate (FFD) sites as compared to intronic SNPs (iSNPs).

**Results:**

The identities of the ancestral bases and the direction of mutations were inferred from human-chimpanzee genomic alignment. After correction for background nucleotide composition, excess of A→G over the complementary T→C polymorphisms, which was observed previously and can be explained by TCR, was confirmed in FFD SNPs and iSNPs. However, when SNPs were separately examined according to whether they mapped to a CpG dinucleotide or not, an excess of C→T over G→A polymorphisms was found in non-CpG site FFD SNPs but was absent from iSNPs and CpG site FFD SNPs.

**Conclusion:**

The genome-wide discrepancy of human FFD SNPs provides novel evidence for widespread selective pressure due to functional effects of sSNPs. The similar asymmetry pattern of FFD SNPs and iSNPs that map to a CpG can be explained by transcription-coupled mechanisms, including TCR and transcription-coupled mutation. Because of the hypermutability of CpG sites, more CpG site FFD SNPs are relatively younger and have confronted less selection effect than non-CpG FFD SNPs, which can explain the asymmetric discrepancy of CpG site FFD SNPs vs. non-CpG site FFD SNPs.

## Background

Single-nucleotide polymorphisms (SNPs) involve two complementary base substitutions, one on each DNA strand. Where the two DNA strands are functionally distinct (such as in transcribed sequences), the two complementary substitutions may not occur with equal frequency on each strand[1], due to transcription-related mutation/repair mechanisms or selective pressure from functional effects on mRNA. A↔G vs. C↔T asymmetry in the two DNA strands is well known to exist in prokaryotes[2]. In the human, there is an excess of C↔T over G↔A in mutations causing Mendelian disorders[3] while excess of A→G substitutions in the sense strand of transcribed intronic sequences was found when comparing a ~1.5 Mb region of human chromosome 7 to its chimpanzee orthologue[4]. Both reports attributed the bias to transcription-coupled repair (TCR), and further support for transcription-coupled effect has been provided by the correlation between strand bias in nucleotide composition of transcribed sequences with transcription levels[5]. However, the conflicting results observed within coding and intronic sequences have not been explored further. It is highly unlikely that TCR distinguishes between exons and introns. Furthermore, our current knowledge of TCR[6,7] suggest that its action would affect the proportion of A→G vs. T→C mutations, but should not affect other mutations. An alternative explanation for the observed discrepancy between exons and introns is that synonymous exonic substitutions in mammals may be under non-trivial selective pressures, as has been suggested by some recent studies[8,9]. An important effect of synonymous coding mutations is the association with gene splicing[10,11]. In humans, evidence of selection on synonymous variations may have a profound effect on how we view the role of synonymous variations in genetic disease and phenotypic variability. Further research is needed besides these studies: the analysis of disease-causing mutations[3] required assumptions about likelihood of coming to clinical attention based on chemical differences between substituted amino acids, while the work on intronic sequences[4] was confined to a single ~1.5 Mb region and the genome-wide applicability of the results remains to be proven. Neither study explored differences between introns and exons to distinguish mutation/repair effects from alterations in RNA function. To our knowledge, strand asymmetry in human SNPs has not been fully examined for possible clues about the mutational mechanisms that created them and/or their potential functional significance. We therefore undertook a systematic examination of human coding SNPs in the fourfold degenerate (FFD) codon site and a random sample of intronic SNPs (iSNPs) for strand asymmetry between A↔G and C↔T polymorphisms.

## Results

The identities of the ancestral bases and the direction of mutations were inferred from human-chimpanzee genomic alignment. To avoid bias from amino acid composition in the third codon position, only FFD SNPs were included in the analysis. On this basis, from the full list of Perlegen validated SNPs, 2,374 FFD SNPs involving A↔G or C↔T polymorphisms were identified for further investigation (Table 1). To increase the statistical power of this study, a larger number of iSNPs were included in the analysis. As edges of introns are known to be under selective constraint[8,12,13], all iSNPs investigated were chosen to be more than 200 bp from each intronic end. In addition, first introns have specific substitution patterns because they are enriched for CpG islands[8] which, being unmethylated[14], are not hypermutable. Also, iSNPs in first introns may be under purifying selection[8,12,13]. Therefore, iSNPs in first introns were not included in the subset.

**Figure 1:**
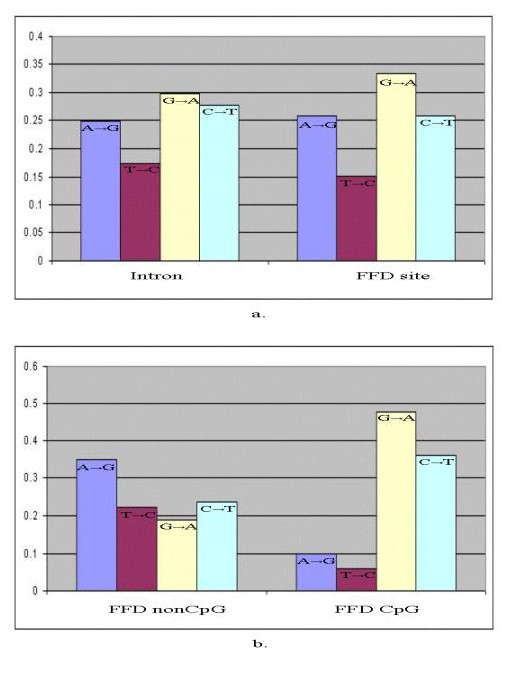
The proportion of each type of A↔G and C↔T substitution (the ancestral allele vs. the recent allele of 7,239 iSNPs and 2,374 FFD SNPs. (a) The proportion of each type of substitution corrected by nucleotide compositions. (b) The FFD SNP distribution corrected by FFD codon compositions. The non-CpG site sSNP distribution is corrected by four types of FFD CpG codons, i.e. NDA, NNT|H, NDG, and NNC|H (D represents A or G or T, and H represents A or C or T). FFD CpG site SNP distribution is corrected by four types of FFD CpG codon compositons, i.e., NCA, NNT|G, NCG, and NNC|G.

To control the observed substitution rates for background nucleotide composition (see Methods), the nucleotide content was determined for all known human intronic sites and FFD sites of coding regions (Table 2). After the background correction, a large excess of A→G polymorphisms over the complementary T→C was found in both iSNPs, χ^2 ^= 122.9, *v *= 1, p < 0.001, ratio (95%CI) = 1.44 (1.35, 1.54), and FFD SNPs, χ^2 ^= 52.7, *v *= 1, p < 0.001, ratio (95%CI) = 1.71 (1.48, 1.98) (Fig 1a). An excess of G→A changes over C→T was also observed in iSNPs, χ^2 ^= 5.0, *v *= 1, p = 0.025, ratio (95%CI) = 1.08 (1.01, 1.15) and, more dramatically, at FFD SNPs: χ^2 ^= 27.2, *v *= 1, p < 0.001, ratio (95%CI) = 1.30 (1.18, 1.43). We thus confirm, genome-wide and within *Homo Sapiens*, the strand bias in substitution rates, which has been found on a human chr. 7 region when compared to chimpanzee[4]. The excess of A→G polymorphisms resulting in iSNPs is concordant with the finding by Green et al [4]. This result can be explained by differential effect of TCR on transcribed and untranscribed DNA strands of genes.

In order to investigate the effect of hypermutable CpG dinucleotides, and to correct for their excess in exons over introns (Table 1), SNPs were next analyzed separately according to whether or not the polymorphism occurred within a CpG site. The hypermutability of CpG dinucleotides is well documented and results from methylation-induced deamination of 5-methyl cytosine[15]. If the deamination occurs on the sense strand, it results in [C→T]pG; if the cytosine deamination takes place on the antisense strand, it produces a Cp [G→A] on the sense strand. Thus, A SNP at a CpG site has the pattern of YpG or CpR (Y represents C or T, and R represents A or G). In introns, the mutational asymmetry does not differ between CpG and non-CpG sites (Table 1). Unlike iSNPs, a dramatic difference between CpG and non-CpG sites was noted in FFD SNPs. After correction for the codon compositions, different asymmetry pattern of G→A vs. C→T between non-CpG sites and CpG sites was noticed (Table 3, Fig 1b). Excess C→T over G→A can be seen in non-CpG FFD SNPs, but not CpG FFD SNPs. Because this finding is present in exons but absent in introns, it is very unlikely that it can be explained by any transcription-related mutational and/or repair mechanism, but suggests selective pressure due to effects on the function of the mature transcript.

An obvious example of such an effect is the creation of an AT dinuclotide by a G→A mutation in a FFD site when a T is the first nucleotide of the next codon. AU dinucleotides are known to be targets of RNaseL endonucleolytic cleavage[16]. A|U dinucleotides at synonymous dicodon boundaries could allow more efficient 3'-5' degradation by endonucleolytic cleavage[17] and, consequently, drive purifying selection. Thus, our interpretation makes the prediction of fewer than expected G→A polymorphisms at FFD sites preceding a codon with a T in the first position. Our analysis indeed shows a dramatic deficit of G→A polymorphisms that occur before a codon that starts with a T (Table 4).

## Discussions and conclusion

It is of great interest that the C→T excess over the complementary G→A in non-CpG FFD SNPs is not seen in iSNPs or FFD SNPs that are part of a CpG. As iSNPs and FFD SNPs should confront the same transcription-coupled mechanisms, including TCR and transcription-coupled mutation (TCM)[18], the C→T excess of FFD SNPs must be driven by mechanisms other than mutational/repair factors. Alternatively, biologically significant effects of synonymous SNPs (sSNPs) on aspects of RNA function other than protein coding may exist and be subject to selective pressures. Unlike lower organisms, it is still contentious whether selection for translational efficiency does[19,20] or does not [21-24] play a major role in shaping codon usage (and therefore sSNP frequencies) in mammals. There is little variation in iso-acceptor tRNA gene numbers and the population sizes are likely too low to reflect very weak selective pressures[23]. On the other hand, translation may be affected by RNA secondary structure which, like splicing, mRNA stability, or other less well understood RNA functions, may be significantly altered by single-nucleotide changes. Such mechanisms have recently been suggested in a few studies[8,9,25]. If sSNPs do have such biological effects, there is evidence to suggest that changes in mRNA secondary structure are likely to play an important role in mediating them[25,26]. Given the evidence of compromised mRNA stability in the presence of A|T dinucleotides at dicodon boundaries [16,17], G→A polymorphisms at FFD sites may have deleterious effects that C→T does not, thus creating selection pressure that favors C→T if the next codon begins with a T. In this report we show that this is indeed the case.

The different asymmetry pattern between non-CpG and CpG sites can be attributed to the hypermutability of the latter[27]. The effects of selection on the observed mutation patterns are most pronounced in relatively slowly mutating, non-CpG sites. Because of the hypermutability of CpG sites, more CpG site FFD SNPs are younger and have confronted less selective pressure than non-CpG FFD SNPs. For the same selection effect on A|T dinucleotides, A→G polymorphism may also confront more selection pressure than T→C, which can also explain why the A→G excess is not significantly different in FFD non-CpG and intronic CpG sites.

In conclusion, we confirm the genome-wide excess of A→G over T→C mutations previously reported in a small region of chr. 7 [4], a finding that points to TCR as an important factor in human mutagenesis. More importantly, our analysis of FFD SNPs clearly suggests a mechanism that operates differentially in intronic vs. exonic sequences. We propose that selective pressure related to changes in mRNA stability is the most likely explanation. In view of the balance between selective and mutational pressures, we provide satisfactory explanation for the previous contradictory findings of mutation rates in humans [3,4,28]. Our finding further highlights the importance of not overlooking potential function by the sSNPs, which may not be as selectively neutral as is generally thought[29], an important consideration given the expected wealth of complex-disease association data to come out of the new genotyping technologies.

## Methods

### SNP information collection

Considering the possibility that some SNPs recorded by NCBI dbSNP database may not be reliable and result from DNA sequencing errors, we performed the investigation using the Perlegen dataset of DNA variation genotyping[30,31]. The SNPs were all identically ascertained by microarray resequencing of the genome, and verified in multiple populations. Only single nucleotide polymorphisms with two alleles were included. SNPs in sex chromosomes were not included in this study. Reference sequences of the SNPs in 22 pairs of human autosomes were bulk-downloaded from the NCBI dbSNP database build 124[32].

### The orientation of SNP reference sequence

The dbSNP reference sequences of iSNPs can not be aligned with mRNA sequence directly. Some FFD SNP reference sequences have intronic sequence included, and some genes have different mRNA transcripts from alternative splicing. Therefore, instead of aligning SNP sequence with mRNA sequence, we wrote Java scripts to determine the orientation of a dbSNP reference sequence in the DNA coding strand. The corresponding NCBI genome DNA contig sequence was first downloaded from the NCBI reference sequences[33]. Then, a SNP reference sequence was aligned with the contig sequence around the SNP contig position and the orientation in the contig sequence was determined. The orientation of mRNA sequence in the same contig sequence was acquired from the annotation of dbSNP. Based on these two orientations, the orientational relation of SNP reference sequence and mRNA sequence was known. The corresponding nucleotide polymorphism in the DNA coding strand were determined consequently.

### Correction for nucleotide or codon compositions

In order to determine the relative rates of each substitution, the observed counts were corrected for the background frequencies of nucleotides or codons. Both the intronic and FFD nucleotide compositions were acquired from the 14,029 genes annotated by the CCDS dababase[34,35]. For background intronic nucleotide compositions, the first introns as well as the first and last 200 bp of each intron were excluded. As an example of correction, for A→G polymorphism, the observed number (N_A→G_) corrected by the frequency of adenine (P_A_) was calculated as:

PA→G=NA→G/PANA→G/PA+NT→C/PT+NG→A/PG+NC→T/PC
 MathType@MTEF@5@5@+=feaafiart1ev1aaatCvAUfKttLearuWrP9MDH5MBPbIqV92AaeXatLxBI9gBaebbnrfifHhDYfgasaacH8akY=wiFfYdH8Gipec8Eeeu0xXdbba9frFj0=OqFfea0dXdd9vqai=hGuQ8kuc9pgc9s8qqaq=dirpe0xb9q8qiLsFr0=vr0=vr0dc8meaabaqaciaacaGaaeqabaqabeGadaaakeaacqqGqbaudaWgaaWcbaGaeeyqaeKaeyOKH4Qaee4raCeabeaakiabg2da9maalaaabaGaeeOta40aaSbaaSqaaiabbgeabjabgkziUkabbEeahbqabaGccqGGVaWlcqqGqbaudaWgaaWcbaGaeeyqaeeabeaaaOqaaiabb6eaonaaBaaaleaacqqGbbqqcqGHsgIRcqqGhbWraeqaaOGaei4la8Iaeeiuaa1aaSbaaSqaaiabbgeabbqabaGccqGHRaWkcqqGobGtdaWgaaWcbaGaeeivaqLaeyOKH4Qaee4qameabeaakiabc+caViabbcfaqnaaBaaaleaacqqGubavaeqaaOGaey4kaSIaeeOta40aaSbaaSqaaiabbEeahjabgkziUkabbgeabbqabaGccqGGVaWlcqqGqbaudaWgaaWcbaGaee4raCeabeaakiabgUcaRiabb6eaonaaBaaaleaacqqGdbWqcqGHsgIRcqqGubavaeqaaOGaei4la8Iaeeiuaa1aaSbaaSqaaiabboeadbqabaaaaaaa@61A8@

The corrected proportions of each type of polymorphisms within the A↔G-C↔T pair were calculated in the same way. For the computation of the asymmetry ratio of complementry polymorphism, such as A→G vs. T→C,

RatioA→G vs. T→C=NA→G/Genome-count (A)NT→C/Genome-count (T)
 MathType@MTEF@5@5@+=feaafiart1ev1aaatCvAUfKttLearuWrP9MDH5MBPbIqV92AaeXatLxBI9gBaebbnrfifHhDYfgasaacH8akY=wiFfYdH8Gipec8Eeeu0xXdbba9frFj0=OqFfea0dXdd9vqai=hGuQ8kuc9pgc9s8qqaq=dirpe0xb9q8qiLsFr0=vr0=vr0dc8meaabaqaciaacaGaaeqabaqabeGadaaakeaacqqGsbGucqqGHbqycqqG0baDcqqGPbqAcqqGVbWBdaWgaaWcbaGaeeyqaeKaeyOKH4Qaee4raCKaeeiiaaIaeeODayNaee4CamNaeeOla4IaeeiiaaIaeeivaqLaeyOKH4Qaee4qameabeaakiabg2da9maalaaabaGaeeOta40aaSbaaSqaaiabbgeabjabgkziUkabbEeahbqabaGccqGGVaWlcqqGhbWrcqqGLbqzcqqGUbGBcqqGVbWBcqqGTbqBcqqGLbqzcqqGTaqlcqqGJbWycqqGVbWBcqqG1bqDcqqGUbGBcqqG0baDcqqGGaaicqGGOaakcqqGbbqqcqGGPaqkaeaacqqGobGtdaWgaaWcbaGaeeivaqLaeyOKH4Qaee4qameabeaakiabc+caViabbEeahjabbwgaLjabb6gaUjabb+gaVjabb2gaTjabbwgaLjabb2caTiabbogaJjabb+gaVjabbwha1jabb6gaUjabbsha0jabbccaGiabcIcaOiabbsfaujabcMcaPaaaaaa@7566@

The 95% CI was computed by logistic regression analysis.

## Competing interests

The author(s) declare that they have no competing interests.

## Authors' contributions

HQQ carried out most of the implementation and analysis, and drafted the manuscript. SGL participated in the SNP database mining. FG wrote the JAVA scripts. JM participated in the study design, the SNP database mining, and the manuscript revision. CP conceived of the study, participated in its design and coordination, and participated in preparation of the manuscript. All authors read and approved the final manuscript.

## Figures and Tables

**Table 1:** The asymmetry pattern of A↔G and C↔T iSNPs and FFD SNPs

**Substitution type**	**A→G**	**T→C**	**G→A**	**C→T**	**Total**
**iSNP**					
**Non-CpG site**	1411 (19.5%)	1030 (14.2%)	1200 (16.6%)	1052 (14.5%)	4693 (64.8%)
**CpG site**	678 (9.4%)	561 (7.8%)	664 (9.2%)	643 (8.9%)	2546 (35.3%)
Non-CpG vs. CpG*	χ^2 ^= 3.2, *v *= 1, p = 0.074		χ^2 ^= 2.1, *v *= 1, p = 0.151		
**Total**	2089 (28.9%)	1591 (22.0%)	1864 (25.8%)	1695 (23.4%)	7239 (100%)
**FFD SNPs**					
**Non-CpG site**	148(6.2%)	127(5.3%)	207(8.7%)	307(12.9%)	789(33.2%)
**CpG site**	316(13.3%)	163(6.9%)	654(27.5%)	452(19.0%)	1585(66.8%)
Non-CpG vs. CpG*	χ^2 ^= 10.9, *v *= 1, p = 0.001		χ^2 ^= 50.1, *v *= 1, p < 0.001		
**Total**	464(19.5%)	290(12.2%)	861(36.3%)	759(32.0%)	2374(100.0%)

* χ^2 ^test of the difference of complementary substitution between non-CpG site and CpG site.

**Table 2:** The nucleotide composition of human genome introns and FFD codons

**Region**	**A**	**T**	**G**	**C**	**Total**
Intron*	126,530,426 (28.0%)	139,073,101 (30.7%)	94,268,606 (20.8%)	92,418,338 (20.4%)	452,290,471 (100.0%)
**FFD codons**	575,500 (19.5%)	615,324 (20.8%)	822,103 (27.8%)	939,137 (31.8%)	2,952,064 (100.0%)

* The first introns as well as the first and last 200 bp of each intron were excluded;

**Table 3:** The proportions of A→G and C→T FFD substitutions corrected by codon compositions

**FFD SNPs**	**Substitution type**	**Observed number**	Codon type*	**Codon count**	**Corrected proportion**	**χ^2 ^test**	**Asymmetry ratio (95%CI)**
**Non-CpG site**	A→G	148	NDA	265,987	0.350	χ^2 ^= 13.6, *v *= 1, p < 0.001	A→G vs. T→C 1.56 (1.23, 1.97)
	T→C	127	NNT|H	355,455	0.225		
	G→A	207	NDG	689,990	0.189	χ^2 ^= 6.2, *v *= 1, p = 0.013	C→T vs. G→A 1.25 (1.05, 1.49)
	C→T	307	NNC|H	818,126	0.236		
**CpG site**	A→G	316	NCA	309,513	0.099	χ^2 ^= 26.0, *v *= 1, p < 0.001	A→G vs. T→C 1.63 (1.35, 1.97)
	T→C	163	NNT|G	259,869	0.061		
	G→A	654	NCG	132,113	0.479	χ^2 ^= 21.3, *v *= 1, p < 0.001	C→T vs. G→A 0.75 (0.67, 0.85)
	C→T	452	NNC|G	121,011	0.361		

* N represents A or C or G or T, D represents A or G or T, and H represents A or C or T

**Table 4:** Decreased FFD SNPs at A|T dinucleotides

**FFD SNPs**	**Substitution type**	**|nonT**	**|T**	**χ^2 ^test**
**Non-CpG site**	G→A	161	46	χ^2 ^= 7.9, *v *= 1, p = 0.005
	A→G	95	53	
**CpG site**	G→A	505	149	χ^2 ^= 19.1, *v *= 1, p < 0.001
	A→G	202	114	
